# Coaxial Printing of Silicone Elastomer Composite Fibers for Stretchable and Wearable Piezoresistive Sensors

**DOI:** 10.3390/polym11040666

**Published:** 2019-04-11

**Authors:** Zhenhua Tang, Shuhai Jia, Xuesong Shi, Bo Li, Chenghao Zhou

**Affiliations:** School of Mechanical Engineering, Xi’an Jiaotong University, Xi’an 710049, China; zhtangy@163.com (Z.T.); xuesongshisxs@163.com (X.S.); polee00@163.com (B.L.); francklinson@163.com (C.Z.)

**Keywords:** coaxial printing, carbon nanotube, silicone elastomer, strain sensor, human motion monitoring

## Abstract

Despite the tremendous efforts dedicated to developing various wearable piezoresistive sensors with sufficient stretchability and high sensitivity, challenges remain pertaining to fabrication scalability, cost, and efficiency. In this study, a facile, scalable, and low-cost coaxial printing strategy is employed to fabricate stretchable and flexible fibers with a core–sheath structure for wearable strain sensors. The highly viscous silica-modified silicone elastomer solution is used to print the insulating sheath layer, and the silicone elastomer solutions containing multi-walled carbon nanotubes (CNTs) are used as the core inks to print the conductive inner layer. With the addition of silica powders as viscosifiers, silica-filled silicone ink (sheath ink) converts to printable ink. The dimensions of the printed coaxial fibers can be flexibly controlled via adjusting the extrusion pressure of the inks. In addition, the electro-mechanical responses of the fiber-shaped strain sensors are investigated. The printed stretchable and wearable fiber-like CNT-based strain sensor exhibits outstanding sensitivities with gauge factors (GFs) of 1.4 to 2.5 × 10^6^, a large stretchability of 150%, and excellent waterproof performance. Furthermore, the sensor can detect a strain of 0.1% and showed stable responses for over 15,000 cycles (high durability). The printed fiber-shaped sensor demonstrated capabilities of detecting and differentiating human joint movements and monitoring balloon inflation. These results obtained demonstrate that the one-step printed fiber-like strain sensors have potential applications in wearable devices, soft robotics, and electronic skins.

## 1. Introduction

Soft and flexible piezoresistive sensors, as a key component of soft electronic devices, have recently become prevalent in various research fields, such as soft robotics, wearable electronics, healthy monitoring, and human–machine interfaces [1]. In particular, stretchable fiber-based sensors, which are expected to be flexible, wearable, and light-weight, are promising as a platform for wearable electronic devices [2]. Furthermore, a fibrous or wire-shaped device can be easily integrated into stretchable fabrics to fulfill a more practical demand of wearable electronics in our daily life [3]. Therefore, tremendous efforts have been made to develop fiber-like piezoresistive sensors [4,5,6,7]. 

Recently, conductive materials such as carbon nanotubes (CNTs) [8], graphene [9], liquid alloy [10], and metal nanowire solutions [11] infiltrated into or coated on stretchable elastomer fibers have been widely used to fabricate fiber-shaped sensors. For example, Cao et al. [11] demonstrated a silver nanowire/polyurethane composite fiber sensor with high sensitivity, but the fabricate method is complex. Zhang et al. [12] fabricated a highly stretchable conductive fiber by dip-coating a layer of liquid metal on silicone elastomer filaments, but the separation between the surface layer and the filament as the fiber bends, stretches, and shrinks is inevitable. To obtain a high-performance wire-like strain sensor, Boland et al. reported a simple method to fabricate graphene-infused elastic bands to produce highly stretchable and sensitive strain sensors [9]. On the other hand, soft and elastic tubes encapsulating conductive fillers have also been used to fabricate fiber-like piezoresistive sensors. For instance, conductive stretchable fibers were fabricated by injecting liquid alloys into elastic polymer tubes [10]. Although large stretchability was achieved, the injection method and the liquid leakage may limit the practical application of this type of fiber-like sensor. Luo et al. [13] filled expanded graphite into an elastic rubber tube to fabricate a high-performance stretchable tubular conductor, but this fabrication strategy is uncontrollable. Zhou et al. [14] combined the wet-spinning approach with a post-treatment process to prepare thermoplastic elastomer-wrapped CNT fiber-like strain sensors, which exhibited high sensitivity, high stretchability, and high linearity, but the fabrication process was complex and time-consuming. In our previous study [5,15], CNT-based coaxial fibers were fabricated via a one-step wet-spinning assembly approach. The sheath layer and core layer of the fibers were pure silicone elastomer and CNT-filled silicone elastic composite, respectively. Due to the elastic nature of silicone elastomer, the stretchability of the coaxial fiber was above 500%. Moreover, the insulating property of the sheath layer of the coaxial fiber avoided the risk of short-circuiting. Various conductive materials and fabrication methods have been employed to fabricate fiber-based piezoresistive sensors. However, most conducting fibers in previous studies were fabricated by physically deposited fillers or solution-coated conducting materials [7,8,9,12]. The common methods such as dip-coating, spray-coating, and a layer-by-layer assembly method have some limitations, such as complex processes, high costs, and manual interventions. Moreover, the conductive surfaces of the aforementioned fibers being exposed and the risk of short-circuiting when used as strain sensors needed to be considered [4,6,7,8,9,12]. The effect of sweat and water in the vicinity on the sensors are also challenges for practical applications. Therefore, the development of wearable and waterproof fiber-like sensors via a simple, efficient, and scalable fabrication process still needs to be addressed. Direct ink writing (DIW) is one of the 3D printing techniques that has drawn much attention, due to its simple printing mechanisms, low cost, and large-scale production. DIW has been applied to print sensors, actuators, energy storage devices, and so on [16,17,18]. For example, Wang et al. [19] fabricated CNT/polydimethylsiloxane(PDMS) strain sensors by layer-by-layer printing CNT dispersion on a PDMS substrate. Kim et al. [20] employed the DIW technique to fabricate eutectic gallium–indium (EGaIn)-based soft sensors. More recently, Cheng et al. [21] fabricated hybrid solid-state electrolytes by using the DIW technique without any additional processing steps. These explorations show that DIW has huge potential in fabricating advanced devices with low-cost, high-efficiency, and mass production properties. 

Here, we employed DIW printing technology to print coaxial CNT-based polymeric composites for superelastic fiber-shaped piezoresistive sensors. The core layer ink was composed of CNTs and silicone elastomer solution, and the sheath layer ink was a viscoelastic silica-filled silicone elastomer mixture. Silica nanoparticles were added into the silicone elastomer solutions to modify their rheological properties and reinforce the mechanical properties. A coaxial nozzle was used in this method, of which the out nozzle and inner nozzle were used to extrude the silica-filled silicone elastomer and CNT/silicone inks, respectively. Appropriate process parameters were selected to ensure a successful printing process. The printed coaxial fibers exhibited excellent mechanical and electrical properties, which could be used as stretchable and wearable piezorisitive sensors. The printed fiber-like sensor exhibited an ultrahigh sensitivity (gauge factor (GF) of 2.5 × 10^6^ at a strain of 90–150%), a large stretchability (150%), and excellent durability and repeatability (over 15,000 cycles). Furthermore, we demonstrated the waterproof property and the detection of various human motions with the printed sensor. The combination of one-dimensional (1D) coaxial fiber design and the easy, low-cost, and scalable DIW printing technology can offer a promising solution for wearable and high-performance electronic devices.

## 2. Materials and Methods

### 2.1. Materials and Characterizations

Unless otherwise specified, all materials were used as received. Multi-walled carbon nanotubes (CNTs, average diameter: 12 nm, length: 10–30 µm, and purity: >98%) were purchased from Chengdu Organic Chemicals Co., Ltd. of the Chinese Academy of Science, Chengdu, China. Silica nanoparticles (Si NPs) with 7–40 nm particle size and 300 m^2^/g specific surface area were supplied by Shanghai Aladdin Biochemical Technology Co., Ltd. Commercially available silicone elastomer Ecoflex 0030 was purchased from Smooth-On (Macungie, PA, USA). The surface morphology of CNTs was characterized by a field-emission scanning electron microscope (SEM) (Zeiss GenimiSEM 500, Oberkochen, Germany). The quality of CNTs was analyzed by Raman spectroscopy HR800 (Jobin Yvon Horiba, Paris, France) with a laser excitation wavelength of 633 nm. The sizes of the fibers were obtained using an optical microscope (GP–300C, Kunshan Gaopin Precision Instrument Co., Ltd., Kunshan, China).

### 2.2. Preparation of the Printable Inks

Homogeneous printable inks were prepared as follows: The sheath silicone elastomeric ink was synthesized by mixing 1 Ecoflex 0030 Part A to 1 Part B with a certain amount of Si NP, which was added as a rheological modifier [22]. To achieve appropriate rheological properties for a 3D printing ink, the Si NP content of the sheath ink was optimized to be 5.5 wt %. The core inks were prepared as follows: Ecoflex 0030 Part A and B were mixed at a 1:1 ratio, followed by the addition of a proper amount of CNT powders. The inks were mixed using a planetary centrifugal mixer (HM800, Shenzhen Hasai Technology Co., Ltd., Shenzhen, China) at 2000 rpm for 5 min. 

### 2.3. Rheological Measurements

The rheological properties of the inks were characterized using a stress-controlled rheometer (MCR302, Anton Paar, Graz, Austria) with a 25-mm diameter parallel plate at room temperature. The viscosity of the inks was measured through a shear-rate sweep from 0.1 to 100 s^−1^. Dynamic stress sweeps were performed at a fixed frequency of 1 Hz to get storage modulus (G′) and loss modulus (G″) as a function of the shear stress sweep from 0.1 to 1000 Pa.

### 2.4. Coaxial Printing

Coaxial printing was carried out using a homemade 3D printing system, which consisted of a computer-controlled 3-axis movement platform. All printing paths were determined using G-code commands, which were generated by commercial software (CuraEngine, Geldermalsen, The Netherlands) from designed 3D models (SolidWorks, MA, USA). Two 20 mL pneumatic syringes were used to store the core and sheath inks individually. An air cylinder connected to an air compressor and two pressure regulators were used to provide the appropriate pressure to extrude the ink through the coaxial nozzle. The inks were extruded through a coaxial nozzle at a distance of 1.5 mm from the substrate. The coaxial printing nozzle was fabricated by inserting a 19 G stainless steel needle (diameter: 0.67 mm) into a 13 G stainless steel needle (diameter: 1.9 mm). The usual printing speed was about 2 mm s^−1^, and the printing pressure of the sheath ink was about 0.64 MPa, which were the optimized parameters. The flow rate of the core ink was controlled by an air pressure governing valve that could flexibly adjust the pressure (range from 10 to 90 kPa) of the air to drive the inks. After printing, the final obtained fibers were fully dried in an oven at 40 °C for 1 h. Unless otherwise specified, the samples for all experiments in this research were printed at 70 kPa and 0.64 MPa for core ink and sheath ink, respectively.

### 2.5. Elecreomechanical Response Measurement

Copper wires were connected to the two ends of the printed coaxial fiber as external electrodes with the help of conductive silver paste and silicone adhesive (Sil-Poxy, Smooth-On). The gauge length between the copper wires was 20 mm. The silicone adhesive was used to cover the silver electrodes to avoid mechanical failure between the soft fiber and rigid electrodes. A computer-controlled homemade stretching stage was used to apply the desired strains. Piezoresistive responses of the printed sensors were measured by recording the current at a constant voltage of 1 V. The electrical responses of the sensors were acquired with a data acquisition module (NI USB-6341, National Instruments, Austin, TX, USA) and transmitted to a computer. All experiments in this study were conducted at room temperature (about 24 °C).

## 3. Results

**Figure 1:**
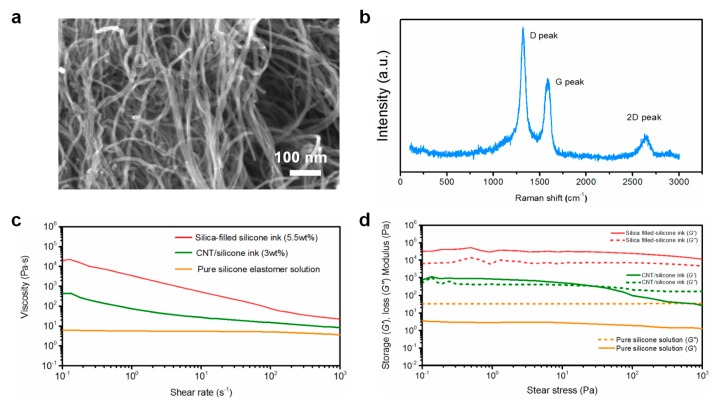
(**a**) SEM image of carbon nanotubes (CNTs). (**b**) Raman spectra for raw CNTs. (**c**) Apparent viscosity as a function of shear rate for various inks. (**d**) Storage modulus (G′) and loss modulus (G″) as a function of shear stress for various inks.

Figure 1a shows the SEM image of the pristine CNT powders, which had an average diameter of 12 nm. Figure 1b shows the Raman spectrum for the raw CNTs. The D peak at 1320 cm^−1^, G peak at 1580 cm^−1^, and the 2D peak at 2640 cm^−1^ were observed. The D peak is attributed to the disorder of the carbonaceous structures. The G peak is associated with the sp^2^ vibration of a perfect graphite crystal [23]. In general, the less disordered the graphite-based systems are, the weaker the intensity of the D peak (relative to the intensity of the G peak) is expected to be. As shown in Figure 1b, the calculated intensity ratio of the D peak to the G peak (*I*_D_/*I*_G_) turned out to be about 1.5, indicating a high level of impurity or defect density in the purchased CNTs. To prepare inks for printing, the rheological properties should be taken into account.Figure 1c shows the curves of viscosity as a function of shear rate for various inks. The apparent viscosity value of pure silicone solutions is less than 10 Pa·s, which is much lower than the printable inks we used, indicating inferior printability. Unlike the pure silicone inks, the addition of CNTs or Si NPs makes the silicone-based composite inks exhibit a remarkable shear-thinning behavior, which is critical for controllable extrusion during printing. Figure 1d shows the storage modulus (G′) and loss modulus (G″) as a function of shear stress for various inks. Storage modulus describes the solidification behavior of the ink in low shear stress conditions, while the loss modulus reflects the liquid-like response. A high storage modulus at low shear stress helped the inks retain their filamentary form after printing. For sheath ink, the content of Si NPs for sheath inks was optimized to be 5.5 wt %, considering the high shape retention at various printing conditions. As shown in Figure 1d, the storage modulus (G′) of pure silicone solution is much lower than its loss modulus (G″), indicating a liquid-like behavior. The storage modulus (G′) of silica-filled silicone inks plateaus above 10^4^ Pa which is much higher than the relevant loss modulus range. These results indicate that silica-filled silicone inks were transformed into a solid-like fluid, due to the formation of a strong silica network at this filler loading, which facilitated the shape retention of the printed patterns. Moreover, the core ink shows a high G′ over 10^3^ Pa, which is higher than G″ in the low shear stress region. This result indicated that the CNT/silicone ink was also transformed into a solid-like fluid, due to the interaction between CNTs and polymer chains. It should be noticed that the G′ and G″ of the sheath ink are much higher than those of the core ink. The higher G′ indicated a stiffer nature of the silica-filled silicone ink, which is desirable for printing self-supported structures without deformation. In fact, owing to the coaxial structural feature of the fiber composite, the higher G′ of the sheath ink was able to lower the requirements for rheological properties of the core ink. That is to say, the rheological properties of the core inks don’t need to meet the printing requirements (shear-thinning and larger storage modulus) in this study. 

**Figure 2:**
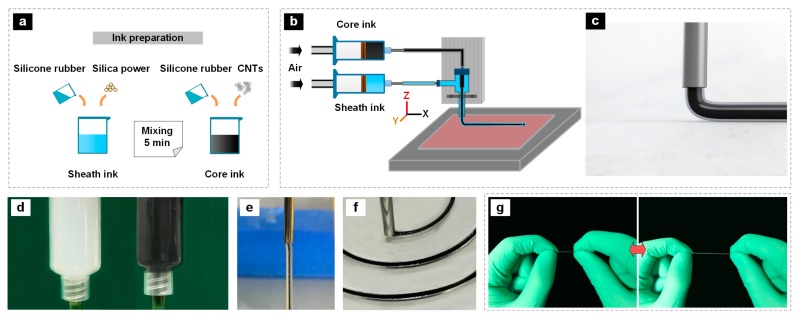
(**a**) Schematic showing the preparation of the printable inks. (**b**) Schematic illustration of the coaxial printing system. (**c**) Schematic illustration of the coaxial printing process and the coaxial structure of the printed fiber. (**d**) Digital optical images of silica-filled silicone ink (**left**) and CNT/silicone ink (**right**). (**e**) Digital optical image of ink-extruding. (**f**) Photograph showing the coaxial printing process. (**g**) Digital optical images of the printed fiber under original (**left**) and stretched (**right**) state, showing the excellent stretchability of the fiber.

Figure 2a briefly shows the preparation process of the silicone-based inks. The silica-filled silicone ink for the sheath layer was formulated by simply mixing Si NPs and silicone solution in a certain weight proportion to create a uniformly dispersed and high-viscosity composite ink. Similarly, the core layer ink was fabricated by mixing CNTs and silicone solution in a certain weight content. The prepared silica-filled silicone ink and CNT/silicone ink were mixed using a planetary centrifugal mixer at 2000 rpm for 5 min. Then the prepared inks were housed in separate syringes (20 mL volume) for coaxial printing. As shown in Figure 2b, the loaded syringes attached with the coaxial nozzle were then mounted onto the specially designed 3D DIW printer. The coaxial printing process is illustrated in Figure 2c. Figure 2d shows the optical images of the loaded syringes with the prepared inks. Figure 2e shows the optical image of the extruding process of the inks through the coaxial nozzle. From this figure, it can be seen that the extruded filament exhibited a clear core–sheath structure and could maintain its shape without breakdown, indicating the printability of the inks. The flow rates of core layer ink and sheath layer ink were separately controlled by air pressure governing valves that could flexibly adjust the injection pressure of air to drive the inks. The movement speed of the nozzle and the printing pressure of the sheath ink were about 2 mm s^−1^ and 0.64 MPa, respectively, which were the optimized parameters. The pressure of the core layer ink was adjusted from 10 to 90 kPa. Figure 2f shows the digital optical image of the printing process of a coaxial fiber. Finally, the printed samples were cured at 40 °C for 1 h and removed from the substrate after cooling down to room temperature. Figure 2g shows that the printed fibers can be stretched to above 100% of tensile strain, demonstrating their high flexibility and stretchability.

**Figure 3:**
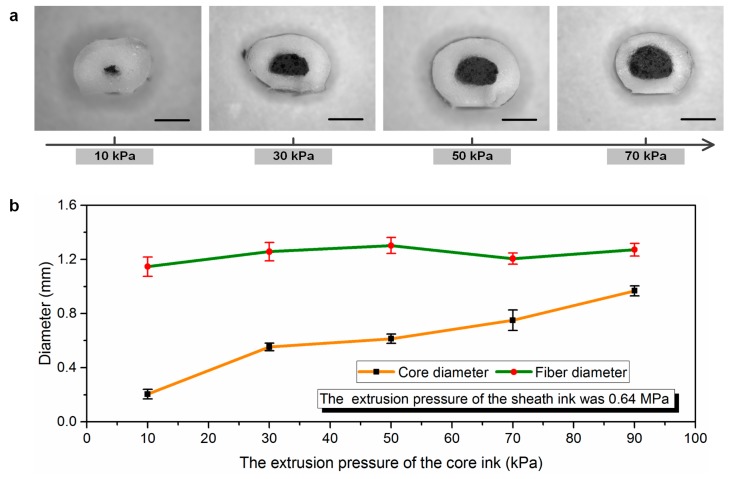
(**a**) Optical microscopy images of the transverse cross-sectional areas of the printed fibers with respect to different injection pressures. The extrusion pressure of the sheath ink remained constant (0.64 MPa). Five samples were measured for each fiber type. All scale bars represent 0.5 mm. (**b**) The whole size of the fiber and the core diameter of the fiber as a function of the inner layer injection pressure.

Figure 3a shows the optical microscopy images of the transverse cross-sectional areas of the printed fibers. From this figure, it can be seen that the printed coaxial fibers consisted of conductive CNT/silicone elastomer composites (black areas), which were encapsulated by elastomeric insulating layers made up of fumed silica-filled silicone elastomer. The cross-section areas were measured form the optical microscopy images with respect to different extrusion pressures. Figure 3b shows the diameter variation of the printed samples under different extrusion pressures of core inks. From this figure, it can be noted that the core diameter of the coaxial fibers increased with the corresponding extrusion pressure. The diameters of the coaxial fibers, however, show only a slight variation, as depicted in Figure 3b. The total diameter of the fiber slightly increased when increasing the core ink injection pressure to approximately 50 kPa, and then the fiber diameter decreased slightly with the increasing pressure. This may be attributed to the thickness of the sheath layer decreasing as the diameter of the core layer increased. The higher the extruding pressure of core ink, the larger the core diameter, but increasing the core ink injection pressure too much would result in the thickness of the sheath layer decreasing quickly. Therefore, the total diameter of the printed fibers exhibited a reducing trend under a certain high extrusion pressure.

**Figure 4:**
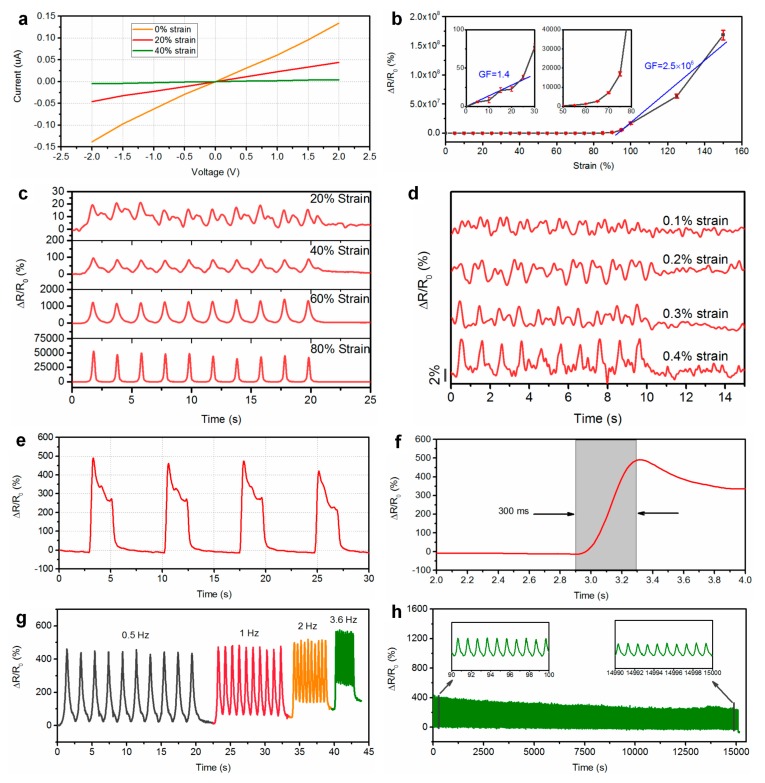
(**a**) Current–voltage (*I–V*) curves of the printed sensor with applied various strains. (**b**) Normalized resistance changes of the sensor under tensile strain. The insets show the normalized resistance changes of the sensor at low strain ranges. (**c**) Dynamic responses of the sensor to repeated strains of 20%, 40%, 60%, and 80%. (**d**) Normalized resistance changes of the sensor under strains of 0.1 to 0.4%. (**e**) Normalized resistance changes of the sensor upon applying a quasi-transient step strain from *ε* = 0% to *ε* = 50%. (**f**) Magnified sensor responses extracted from (**e**) to show the response time. (**g**) Relative resistance changes of the sensor vs. a tensile strain of 50% at frequencies of 0.5, 1, 2, and 3.6 Hz. (**h**) Normalized resistance changes of the sensor under repeated stretching/releasing cycles with 50% strain at a frequency of 1 Hz for 15,000 cycles, demonstrating the durability of the printed sensor. The insets show the normalized resistance changes of the sensor from 90 to 100 s and 1490 to 1500 s.

Figure 4 shows the current–voltage (*I–V*) curves of the printed fiber-like sensor under various static strains, ranging from 0 to 40%. From this figure, it can be noted that the *I–V* curves all appeared to have a linear tendency, indicating the ohmic behavior and constant conductivity of the sensor under static loading. Therefore, the resistance of the device is independent of the applied voltage. The piezoresistive behavior of the sensor was investigated by monitoring the relative changes in electrical resistance for the applied strain. The resistance of the sensor increased with the applied strain, and the electric connection was lost under a strain above 150%. It should be noted that the fracture strain of the coaxial fiber was about 400%. Figure 4b shows the relative changes in electrical resistance (Δ*R*/*R*_0_) of the sensor under various strains. The gauge factor (GF) is a characteristic parameter representing the sensitivity of the sensor and can be calculated from (Δ*R*/*R*_0_)/*ε*, where Δ*R*, *R*_0_, and *ε* denote the change in resistance, initial resistance, and applied strain, respectively. The calculated GFs of the sensor were 1.4 and 2.5 × 10^6^ for strains ranging from 0 to 25% and 90 to 150%, respectively. Figure 4c shows the normalized resistance changes of the sensor under various cyclic strains of 20%, 40%, 60%, and 80%. From this figure, it can be noted that the sensor exhibits a uniform and repeated response according to the applied strain. A shoulder peak pattern was observed during strain releasing. The shoulder peak originates from the competition between the destruction and reconstruction of the conductive networks during the releasing process. A similar behavior has been previously reported [24,25]. Here, the shoulder peaks for larger strain of 60% and 80% were negligible. In addition, as shown in Figure 4d, the sensor could successfully differentiate strain from 0.1% to 0.5%, indicating the capability of detecting subtle strains. The dynamic response of the sensor under a square wave loading signal is shown in Figure 4e. From this figure, it can be seen that the sensor exhibited a consistent change in resistance, indicating excellent repeatability. During stretching, the response of the sensor showed overshooting in response to acceleration. A similar phenomenon has been previously reported for CNT-based polymer composites [5,26]. This behavior may be attributed to the viscoelasticity of the silicone elastomer. Figure 4f shows the response time of the sensor. The response time is estimated to be around 300 ms. If the relay of the measurement system were considered, the actual response time for the sensor itself would be even shorter than this value. For the frequency response test, we applied a strain of 50% at a varying strain rate of 0.5 to 3.6 Hz. Figure 4g shows the relative change of the resistance for the sensor at various frequencies. From this figure, it can be noted that the sensor retained its performance until a strain rate of 3.6 Hz, showing a stable variation in resistance. The relative resistance change slightly increases with an increase of the frequency of the applied strain. This may be attribute to that the higher strain rate causes greater stress in the materials, which leads to an increase in the amplitude of relative resistance change at high frequencies [5]. Moreover, the dynamic durability of the sensor was investigated by monitoring the response of the sensor under cyclic stretching at a strain of 50% at 1 Hz. As shown in Figure 4h, the sensor maintained the sensing performance for 15,000 cycles, indicating that the sensor had a long working life and excellent repeatability. 

**Figure 5:**
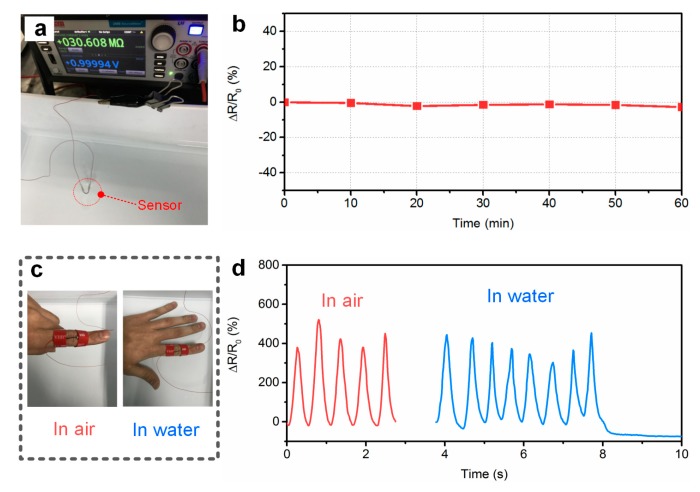
(**a**) The fiber sensor system while soaking in water. (**b**) Relative changes in electrical resistance of the sensor as a function of the soaking time. (**c**) Photographs of the sensor directly attached to the index finger. (**d**) A comparison of the dynamic responses of the printed sensors during finger bending movements in air and water at room temperature.

The stability of the sensor in a wet environment is necessary for practical application. The electrical response of the sensor to water was monitored for one hour by soaking the sensor in water (Figure 5a). Figure 5b shows the relative changes in resistance of the sensor as a function of the soaking time. From this figure, it can be noted that there were no significant changes in the electrical resistance after soaking in water for 60 min. In addition, the sensor was attached onto the back of the index finger by bonding the two ends with a stretchable tape (Figure 5c), and the dynamic response of the sensor in water at room temperature was recorded. The electrical resistance was recorded by the source meter (Model 2450, Keithley, Cleveland, OH, USA). Owing to the flexibility of the sensor, conformal attachment to uneven skin can be achieved. Figure 5d shows the relative changes in resistance of the sensor during the bending motions of the finger in air and water at room temperature. From this figure, it is clearly seen that the dynamic responses of the sensor in air and water were similar, indicating that the sensor exhibited excellent waterproof properties. This can be attributed to the conductive core being fully sealed with hydrophobic silicone rubber, which prevented water molecules from being absorbed into the sensor surface. Therefore, the sheath layer effectively reduced the effect of the water on the sensors.

**Figure 6:**
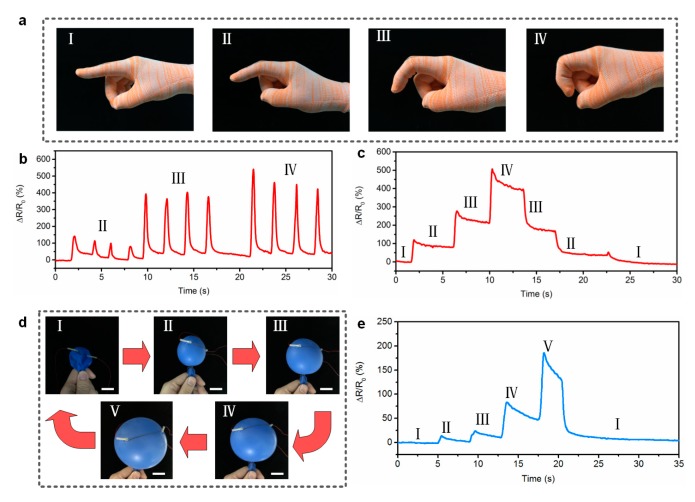
(**a**) Optical images of a sensor woven into a glove at different bending angles. (**b**) Relative changes in resistance for bending/unbending motions of an index finger with various bending angles. (**c**) Electrical resistance responses of the sensor under consecutive step-and-hold tests. (**d**) Photographs of a strain sensor attached to a balloon at various inflating states. All scale bars represent 20 mm. (**e**) Monitoring of tension changes on the balloon surface during inflation.

Furthermore, to demonstrate the potential of the printed fibers as wearable sensors, the fiber-shaped sensor was woven into the index finger of a fabric glove, using a sewing method. Figure 6a shows the photographs of a fiber sensor woven into a glove. Figure 6b shows the relative changes in resistance of the sensor under cyclic bending motions (various bending angles). From this figure, it can be seen that the sensor responded to the motion of the finger quickly and accurately. Moreover, the sensor reliably detected resistance changes, depending on the degree of bending of the finger, and the sensor distinguishably responded to different finger motions. Figure 6c shows dynamic resistance changes of the sensor under consecutive step-and-hold tests. From this figure, it can be seen that the resistance variation increased in a stepwise manner in real time as the bending angle of the finger increased step by step. When the finger recovered to its original state gradually, the resistance decreased according to the bending angles. The above results fully indicate that the as-printed sensor with high sensitivity and excellent stability presents potential applications for monitoring and analyzing human motions.

Thanks to the excellent flexibility and ultra-light weight of the fiber, the sensors could be attached conformally to various uneven surfaces. As a demonstration, the sensor was directly attached to the surface of a flat balloon (Figure 6d). The balloon was blown to expand its surface, while the electrical resistance was recording in real-time. Figure 6e shows the resistance changes of the sensor, depending on the gas volume in the balloon. From this figure, it can be seen that the response curve of the sensor in a stair-like pattern corresponds to the process of the blowing. When the expanding balloon deflated, the electrical resistance reduced. Based on the above results and the wide sensing range and remarkable sensitivity of the sensors, we believe that the printed fiber-like sensor demonstrates its high potential for application as a wearable strain sensor.

## 4. Conclusions

In summary, we demonstrated a simple and efficient strategy to construct a stretchable and flexible fiber-shaped strain sensor with both high sensitivity and large sensing range, through a coaxial printing technique. A series of novel printable silicone elastomer-based inks with shear-thinning behavior was developed. Through the addition of fumed silica powders or CNTs as viscosifiers, silicone elastomer-based inks became adaptable for DIW printing, and coaxial fibers were successfully printed with high-throughput production capability. The printed coaxial fibers exhibited high stretchability and excellent flexibility, which were used as wearable strain sensors. It was demonstrated that the fiber-like strain sensors showed high sensitivity (maximum gauge factor of 2.5 × 10^6^ at 90 to 150% strain), large sensing area (0–150% strain), outstanding durability (15,000 cycles), and excellent waterproof performance. Furthermore, the printed coaxial fiber-based sensors were proven to be capable of detecting and differentiating human joint motions and other curved surface deformations. The one-step coaxial printing and synthesis of the inks in this work could provide inspiration for the development of conformal and readily fabricated lightweight wearable electronics.
